# Pupil dilation reflects the dynamic integration of audiovisual emotional speech

**DOI:** 10.1038/s41598-023-32133-2

**Published:** 2023-04-04

**Authors:** Pablo Arias Sarah, Lars Hall, Ana Saitovitch, Jean-Julien Aucouturier, Monica Zilbovicius, Petter Johansson

**Affiliations:** 1 Lund University Cognitive Science, Lund University, Lund, Sweden; 2 STMS Lab, UMR 9912 (IRCAM/CNRS/SU), Paris, France; 3 School of Neuroscience and Psychology, Glasgow University, Glasgow, UK; 4 U1000 Brain Imaging in Psychiatry, INSERM-CEA, Pediatric Radiology Service, Necker Enfants Malades Hospital, Paris V René Descartes University, Paris, France; 5 Department of Robotics and Automation FEMTO-ST Institute (CNRS/Université de Bourgogne Franche Comté), Besançon, France

**Keywords:** Human behaviour, Emotion

## Abstract

Emotional speech perception is a multisensory process. When speaking with an individual we concurrently integrate the information from their voice and face to decode e.g., their feelings, moods, and emotions. However, the physiological reactions—such as the reflexive dilation of the pupil—associated to these processes remain mostly unknown. That is the aim of the current article, to investigate whether pupillary reactions can index the processes underlying the audiovisual integration of emotional signals. To investigate this question, we used an algorithm able to increase or decrease the smiles seen in a person’s face or heard in their voice, while preserving the temporal synchrony between visual and auditory channels. Using this algorithm, we created congruent and incongruent audiovisual smiles, and investigated participants’ gaze and pupillary reactions to manipulated stimuli. We found that pupil reactions can reflect emotional information mismatch in audiovisual speech. In our data, when participants were explicitly asked to extract emotional information from stimuli, the first fixation within emotionally mismatching areas (i.e., the mouth) triggered pupil dilation. These results reveal that pupil dilation can reflect the dynamic integration of audiovisual emotional speech and provide insights on how these reactions are triggered during stimulus perception.

## Introduction

Emotional speech perception is an audiovisual process. Emotion classification improves when individuals have access to audiovisual cues^1^, because auditory and visual information are integrated jointly during affective decisions^2^. On the one hand, congruent audiovisual signals such as e.g., prosody, semantics, or face articulation, additively improve emotion recognition performance^3^. On the other hand, incongruent audiovisual signals ambiguate and slow down emotion decision processes^4,5^. These audiovisual integration processes seem to be automatic^6^, fast^7,8^ and robuts^9^, because the effects are present even when explicitly instructing participants to ignore one of the two sensory channels, or for high levels of cognitive load. These cognitive mechanisms seem to rely on regions such as the superior temporal gyrus, superior temporal sulcus, amygdala, and bilateral thalamus^1,10,11^. Functional deficits in these areas due to e.g., schizophrena^12^ are known  to impair audiovisual emotional integration^13,14^.

To study audiovisual integration, previous research has mostly studied the cognitive processing of information mismatch. For instance, studies on the classic McGurk effect use mismatching voice and face vocalizations to investigate how audio and visual signals are merged into unified percepts^15–17^. Similarly, information mismatch is used for the study of audiovisual emotions. For instance, the perception of an emotion in one modality is biased by the perception of a mismatching emotion in another modality^6^. Recent findings suggest that the cognitive processing of audiovisual information mismatch relies on general-purpose conflict areas (e.g., anterior cingulate cortex), audiovisual speech conflict areas (e.g., inferior frontal gyrus)^18^, and increases in theta band activity—similar to general purpose conflict mechanisms^19^.

Interestingly, pupil dilation seems to be a good candidate to index information mismatch during the perception of audiovisual emotional stimuli. First, previous findings report that pupil dilation can respond to incongruent semantic vs visual^20^ information, or action vs emotion^21^ information. Second, pupil dilation has been associated with emotional processes. Pupil dilation—under isoluminance conditions—is almost exclusively promoted by norepinephrine release from the locus coeruleus, a brainstem nucleus associated with e.g., implicit emotion processing^22^ or alarm for fear stimuli^23^. In the neuroscientific literature, pupil dilation has even been outlined as a measure to assess arousal and attentional control^24^. In this line, previous studies identified pupillary reactions when e.g., arousing pictures are presented to participants^25^, a mechanism that develops early during infancy^26^ and is recruited even when stimuli are presented outside of conscious awareness^27^. Similarly, arousing vocalizations trigger pupil dilation^28^, a reaction which seems to be both associated with emotion decision processes^29^ and sensitive to emotional load and authenticity^30^. However, to our knowledge, the pupillary reactions triggered by audiovisual emotion information mismatch remain unknown.

In the current study we investigated if pupil dilation can index the processes underlying audiovisual emotion integration. To do this, we investigated pupillary reactions in response to information mismatch. Specifically, we created speech stimuli where vocal and visual emotions were either congruent or incongruent, while controling for the temporal synchrony between visual and auditory channels. To do so, we used a recently developed digital signal processing algorithm able to increase or decrease the smiles seen in a person’s face or heard in their voice, while leaving unchanged all other characteristics of their speech—such as their semantics, intonation or prosody^31^. This digital algorithm is composed of two separate audio and visual pipelines. On the one hand, the auditory model controls the amount of smiles heard in the voice by shifting the first two vocal formants—a critical feature for auditory smile perception^32–34^. On the other hand, the visual model controls the smiles seen in the face by deforming facial morphology and recreating the facial shape and texture of a smile. Importantly, the transformation occurs on a frame-by-frame (or buffer-by-buffer, in the case of audio) basis, which means that transformed stimuli share the same morphology, length, semantic content, speech rate, prosody, identity, luminance, and vary only on their audiovisual smile content.

Previous evidence investigating the causes underlying pupil reactions is mixed: it is not clear whether pupil dilation occurs because of emotion recognition processes or automatically. On the one hand, studies have linked pupil dilation with explicit emotion decision processes^29^ explicit emotion tasks^30^, and high levels of cognitive load^35^. On the other hand, studies have used implicit tasks^25^ and observed reactions even when stimuli were presented outside of conscious awareness^36^. For these reasons, we investigated whether performing an emotion recognition task influenced pupillary reactions. Moreover, because one novel aspect of our work is to use audiovisual speech where voices and faces are in synch, we also investigated the dynamics of the physiological reactions, namely, the interaction between participants’ gaze patterns and subsequent physiological reactions.

In short, in the present study, we aim to use an algorithmic model^31^ to create congruent and incongruent audiovisual smiles and measure participants’ pupillary and gaze reactions during the perception of audiovisual stimuli. Our goal is to investigate (1) if pupil dilation can index information mismatch during emotional speech perception, (2) if performing an emotion extraction task can influence pupillary reactions and (3) investigate the dynamics of physiological reactions.

## Methods

### Stimuli

We asked French speakers to record a set of sentences with a natural tone—as if they were talking to another person—in an audiometric booth, with a black background, using a tripod, an LM400 light, a DPA 44100 omnidirectional microphone and a Sony (HVR-Z5E) camera. We chose nine video recordings (min duration = 5.2 s, max duration = 8.0 s, mean duration = 6.2 s) among the whole recordings, so that stimuli varied in affect (either neutral or positive), speaker (3 females, 1 male; 4 Caucasian), while being intelligible, focused, with fluid speech rate, with a straight gaze to the camera, and at least 5 s long—to allow physiological reactions to happen during stimulus presentation (See the sentences used in SI).

We transformed the nine selected recordings using a recently developed audiovisual smile algorithm^31^. On the one hand, the visual transformation either increases or decreases the visual smiles seen in the face using a pre-learned deformation model and later recreating realistic colours and textures using a Moving Least Square algorithm. On the other hand, the auditory transformation shifts the first two vocal formants of the voice using a piecewise linear frequency warping, a critical feature for auditory smile perception^32^. We validated these algorithmic models in previous research. We showed that the vocal transformation is correctly decoded as smiliness and that it can trigger zygomatic major (the muscle used to smile) activity both in naïve^37^ and congenitally blind^38^ participants. We also validated the visual algorithm on emotional and smiliness scales^31^, and studied the integration between audiovisual channels, e.g., showing that smile information from visual and auditory channels jointly influence emotional rating tasks^31^.

Using these algorithms, we first transformed each video with two video transformations: increase smile and decrease smile. Then, for each transformed video, we transformed audio recordings either increasing or decreasing auditory smiles. This way, we generated a total of 36 stimuli: 18 congruent (where we increase or decrease smiles in both audio and video channels) and 18 incongruent (where we increase the smiles in one channel and decrease them in the other one). You can find stimuli examples in the Supplemental Information. It is crucial to highlight that we used *exactly* the same video/audio transformations to create congruent and incongruent videos. For instance, to create an incongruent version of Recording 1, we increase the smile of the visual channel while decreasing the smile of the auditory channel (or vice-versa). To create the congruent version, we increase the smiles of both channels or decrease the smiles of both channels. Thus, the sound and video used to create both congruent and incongruent stimuli are *exactly* the same, the only thing that varies between congruent and incongruent conditions is the pairing between visual and auditory streams. This allows us to control for the variability intrinsically present in the original recordings such as the identity, semantic content, speech rate, etc. In consequence, an effect in reaction to *congruence* in these stimuli can only be due to the association between visual and auditory channels. For these reasons, between conditions, the stimuli had the same length, the same colours, the same dynamics, the same luminance, the same semantic content, the same prosody, and varied only in the acoustic/visual dimensions of the voice/face manipulated by the audiovisual algorithm.

**Figure 1:**
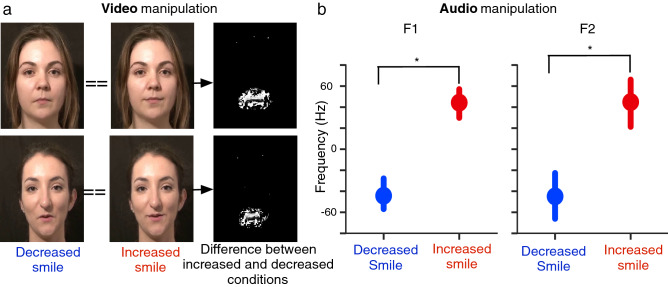
(**a**) Examples of the visual smile transformation. Left: decreased smile-transformation example; Centre: increased smile-transformation example; Right: pixel to pixel Boolean difference between increased and decreased smile conditions, where it can be seen the pixels that have the same (black) or different (white) pixel values for both increased and decreased smile conditions. Note that the differences between conditions are located inside the mouth area (**b**) Change in mean of first formant (left) and second formant (right) frequencies of auditory stimuli grouped by audio transformation (increased or decreased smiles). Stimuli transformed with the increased smiles effect has higher formant frequencies than stimuli transformed with the decreased smile effect; Formants were mean-normalised by the formants of the non-manipulated stimulus. Error bars are 95% confidence intervals on the mean; *Statistically significant differences between distributions (paired t-tests, p < 0.05).

Figure 1-a shows an example of the visual manipulation. Video frames in the left column were transformed to decrease the smiles with the visual transformation; Video frames at the centre were transformed to increase their smiles; The right column presents the pixels where the two transformations differ: pixels in black represent the areas where the pixels in both transformations have exactly the same value, while pixels in shades of grey/white represent the areas where pixels are different. Note that the only differences between manipulated stimuli is located within the mouth area (the only place where pixels are in shades of grey/white). Figure 1-b shows the acoustic effect of the audio transformation. To compute the acoustic effects of the audio transformation, we extracted the mean of the first and second formant frequencies both when increasing and decreasing smiles in the audio recordings. To control for speaker and semantic variability, we normalised formant measures by the formants of the non-manipulated recording. We found that the audio manipulation significantly shifted the first and second formant frequencies (p < 0.0001; paired t-tests), in a way that reflected the acoustic changes characteristic of smiled speech^32^.

### Participants

N = 30 participants (male = 14, female = 16, mean age = 22, min = 18, max = 30) took part in this experiment. All participants gave written consent and were paid at a standard rate of 10 €/h for their participation. All participants reported having no psychological or neurological disorders and no hearing or vision problems. We determined sample size based on previous pupillometry studies investigating pupillary reactions to information mismatch^25^ (N = 34), as well as studies investigating pupillary reactions to affective vocalisations^29,30^ (N = 33; N = 28 participants).

### Procedure

Participants were seated in a windowless room at ~ 65 cm from the computer screen. The artificial lighting was identical and constant between and within participants. The experiment lasted around 25 min per participant. Participants did not know that their gaze and pupil size were monitored during the experiment.

The experiment began with a calibration of the eye tracking device (Tobii Pro X3-120), in which participants had to visually follow a red point moving in the screen. We used the default Tobii Pro X3-120 calibration procedure, which lasted approximately 5 min per participant. Then we proceeded to the two experimental blocks.

The first block was a free viewing paradigm (later refered to as ‘passive task’), where participants were instructed to simply watch the video stimuli. We pseudo-randomised stimuli presentation for each participant by maximizing the distance of appearance of stimuli created by transforming the same original video token. In other words, we never presented manipulations from the same original stimulus one after the other. Each stimulus was preceded by a 1.5 s black screen with a centred fixation cross to ensure that, at the beginning of each trial, participants were looking at the same place in the screen.

After the first block, we displayed on screen instructions to explain the second block. The second block was an emotional task (later referred to as ‘emotion task’), where we aimed to direct participants’ attention to emotional features in the stimuli. To do this, we asked participants to attend to the emotional state of the person in the videos. No explicit judgement was required. Specifically, before each trial, a black panel appeared on the screen with the sentence, “please evaluate the emotional state of the person in the following video”. The panel was followed by a centred fixation cross for 1.5 s and then by stimulus presentation. Stimuli were the same as in block one. Importantly, in order to be able to compare passive and emotional tasks, we did not ask participants a motor response reflecting their emotional perception of the stimuli, as these decision related and motor-preparation processes may influence pupillary reactions^35,39^. Consequently, other than in the instructions, block one and two were strictly identical. The only difference between the passive and the emotion blocks was that in the emotion block, participants attention was directed towards the emotional content in the stimuli.

### Ethics

All experiments were approved by the Institut Européen d’Administration des Affaires (INSEAD) IRB. All methods and experiments were carried out in accordance with the American Psychological Association Ethical Guidelines. All participants gave their informed consent and were debriefed and informed about the purpose of the research immediately after the experiment. Similarly, all persons present in identifying images in Fig. 1, as well as on the Supplemental Video Stimuli, gave their informed consent for publication of their images/videos.

### Gaze data pre-processing

For each stimulus, we defined 4 dynamic Areas of Interest (AOIs): eyes, mouth, rest-of-face (face areas outside the mouth and eyes areas) and background. To extract these areas, we manually coded three rectangular areas in the face which tracked the mouth, the face, and the eyes, across the whole video, for each of the 36 videos. To control for facial movements in the videos, these three areas moved dynamically to always be centred either in the eyes, the mouth or the face. We used these areas to define the AOIs in the analysis stage. To distinguish between saccades and fixations we used an I-VT fixation filter^40^. Raw data were exported and analysed in python 3.7. We excluded all measures where there was no co-occurring presentation of sound and face stimuli (i.e., before and after speakers began/ended talking at the very start and at the very end of each video).

We used two descriptive statistics to describe gaze patterns: fixation duration and number of fixations. To extract these measures, we computed the percentage of number of fixations within a specific AOI, by computing the ratio between the number of fixations to a specific AOI, to the total number of fixations during the trial. Similarly, we computed the percentage of fixation duration as the ratio between the amount of time fixating within a specific AOI and the total fixation time in the trial.

### Pupil size data pre-processing

As for the gaze data pre-processing, we excluded from further analyses all data where there were no co-occurring sound/face signals (at the beginning and at the end of the video sentences). We then used the following preprocessing pipeline to clean pupil data. We measured participants’ left and right pupil size with a Tobii Pro X3-120 (sampling rate: 120 Hz) and coded as NaNs pupil measures where pupil validity—as given by Tobii’s eye tracker—was above a threshold of 4. When only one of the two measure was valid we kept only the valid one. If the two measures were valid, we averaged left and right measures into a single measure. As participants had to actively explore stimuli in dynamic faces during the task, eye movement was important during trials, which added noise to the pupil measure. To reduce this noise, we down-sampled pupil measures to 30 Hz and performed a moving-average with a window size of 15 samples. In the following analyses, whenever we collapsed data across time, we computed the median pupil dilation to control for artefacts in the data caused by saccades, blinks and head movements. To further clean the dataset, we removed all the trials where pupil size was not measured correctly for more than 50% of the trial. With this procedure we excluded 2.8% of the trials. Moreover, we excluded from further analyses all the data (mostly Nans) where pupil size was not computed correctly (e.g., because left pupil size and right pupil size were not measured correctly), a procedure which rejected ~ 7% of the data points.

### Statistical methods

We analysed participants’ gaze and pupil size using GLMMs (Generalized Linear Mixed Models) in R with *RStudio* (1.4.1106) and using the *lmerTest* 3.1-3 package. We report p-values, estimated from hierarchical model comparisons using likelihood ratio^41^ tests and only present models that satisfy the assumption of normality, and statistical validation (significant difference with the nested null model^41^). To test for main effects, we compared models with and without the fixed effect of interest. To test for interactions, we compared models including fixed effects versus models including fixed effects and their interaction. We used participant’s identification number as a random factor. Whenever possible (when models converged) we added random slopes for task and AOI. For post-hoc comparisons we performed paired t-tests corrected for multiple comparisons using Bonferroni corrections.

## Results

### Gaze patterns

Participants’ gaze was classically distributed across face areas. Participants spent 63% of time fixating the eyes, 24% fixating the mouth, 10% fixating the rest of the face, and 1.9% fixating the background.

**Figure 2:**
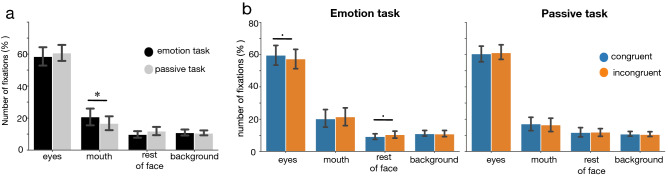
Gaze results. (**a**) Mean number of fixations for each AOI, and for each task. (**b**) Mean number of fixations for each task, for each AOI and for both congruent and incongruent conditions; error bars are 95% Confidence Intervals on the mean; Asterisks indicate statistically significant differences (p < Bonferroni-α = 0.0125), “.”: indicate marginally significant differences (p < 0.05).

To study if gaze patterns were different across tasks, we performed a hierarchical GLMM analysis with *number of fixations* as an outcome, *task* (2 levels: passive, emotional) and *AOI* (4 levels: eyes, mouth, rest of face and background) as predictors, and *participant-id* as random factor with random slopes for task and *AOI* (Fig. 2-a). We found a significant main effect of AOI (χ^2^(3) = 90.5, p = 2.2e−16), no main effect of task (χ^2^(1) = 0, p = 1) and a significant interaction between *AOI* and *task* (χ^2^(3) = 40.4, p = 8.4e^−9^). Bonferroni corrected post-hocs revealed that participants spent 4% (± 0.8 SE) more time fixating the mouth (t(29) = 2.9, p = 0.006) during the emotion task compared to the passive task. For the other AOIs, we observed no difference after Bonferroni correction (Bonferroni-α = 0.0125, eyes: t(29) = 1.2, p = 0.23; rest of face: t(29) = 1.8, p = 0.07, background: t(29) = 0.5, p = 0.58). In short, participants’ gaze patterns seemed to be slightly different between tasks, with a small over exploration of the mouth area during the emotion task. We observed very similar results when using *fixation duration* as an outcome in the GLMM models (see SI Fig. 1-a, SI Supplemental Analysis).

We then investigated if *congruence* affected gaze patterns. To do so, we performed a GLMM analysis to test for effects of *AOI* (4 levels: eyes, mouth, rest of face, background) and *congruence* (2 levels: congruent, incongruent) for each task (Fig. 2-b). We used participant number as a random factor with a random slope for *AOI*. We began by analysing the *number of fixations* during the emotion task (Fig. 2-b). We found no significant main effect of *congruence* (χ^2^(1) = 0, p = 1), a significant main effect of *AOI* (χ^2^(3) = 84.2, p = 2.2e^−16^), and a significant interaction between *AOI* and *congruence* (χ^2^(3) = 10.4, p = 0.01). Paired post-hoc t-tests were not significant after Bonferroni corrections for multiple comparisons (Bonferroni-α = 0.0125; paired t-tests eyes: t(29) = 2.2, p = 0.03; mouth: t(29) = − 1.3, p = 0.17; rest of face: t(29) = − 2.2, p = 0.03; background: t(29) = 0.3, p = 0.7). In the passive task (Fig. 2-b), we found a main effect of *AOI* (χ^2^(3) = 93.1, p = 2.2e−16), no main effect of *congruence* (χ^2^(1) = 0, p = 0.99), and, in contrast to the emotion task, no interaction between *task* and *AOI* (χ^2^(3) = 1.2, p = 0.7). In short, congruence influenced gaze patterns in the emotion task, but differences between conditions were small and did not hold corrections for multiple comparisons. We observed very similar results when using *fixation duration* as an outcome in the models (see SI Fig. 1-b, SI supplemental analysis).

### Pupillometry analysis

**Figure 3:**
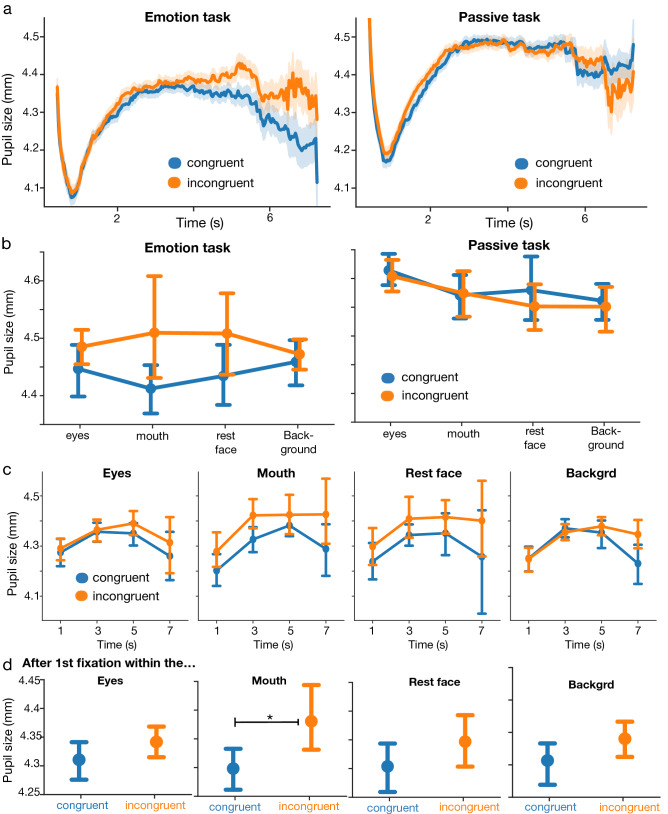
Pupil results (**a**) Mean pupil size time series for both the emotion task (left) and the passive task (right), for both congruent (blue) and incongruent (orange) conditions; Shaded areas represent SEM; (**b**) Mean pupil size for both the emotion and the passive task for both congruent (blue) and incongruent (orange) conditions and for each AOI (**c**) Mean pupil dilation over time for both congruent and incongruent conditions, for each AOI (**d**) Mean pupil size after the first fixation to each AOI for both congruent and incongruent conditions; error bars are 95% confidence intervals on the mean; ‘*’: statistically significant differences between distributions (p < 0.05); ‘.’: marginally significant differences between distributions (p < 0.1).

We then studied if congruence affected pupil dilation. To do this, we first computed median pupil-size time-series for each participant for both congruent and incongruent conditions and for both tasks (Fig. 3-a). Pupil reactions after stimulus presentation began with an initial startle reflex of ~ 700 ms—similar to those observed in previous studies after visual stimulus presentation^42^. Later during the trial, incongruent trials seemed to trigger stronger pupil dilation in the emotion task, although this effect seemed to be absent in the passive task.

To investigate the significance of this effect, we performed a 3-way GLMM analysis using *task* (2 levels: emotion, passive), *congruence* (congruent, incongruent) and *AOI* (4 levels: eyes, mouth, Rest of face, background) as predictors, as well as participant number as a random factor with task as a within subjects random slope (Fig. 3-b). We found a marginally significant main effect of *congruence* (χ^2^(1) = 3.1, p = 0.07), a main effect of *task* (χ^2^(1) = 5.0, p = 0.02) and no main effect of *AOI* (χ^2^(3) = 1.8, p = 0.6). We also found a significant interaction between *congruence* and *task* (χ^2^(1) = 6.5, p = 0.01), but no significant interaction between *congruence* and *AOI* (χ^2^(3) = 2.1, p = 0.5) or between *task* and *AOI* (χ^2^(3) = 2.8, p = 0.4).

To further investigate the *task* x *congruence* interaction we found in the 3-way GLMM, we ran a 2-way GLMM analysis for each task. Specifically, we tested for main effects and interactions of *AOI* and *congruence*, using participant number as a random factor for each task. In the emotion task, we found a main effect of *congruence* (χ^2^(1) = 6.9, p = 0.008), no main effect of *AOI* (χ^2^(3) = 0.17, p = 0.98) and no interaction between *congruence* and *AOI* (χ^2^(3) = 2.4, p = 0.48; Fig. 3-b left). For the best fit model, incongruent trials increased *pupil size* by 0.05 ± 0.02 (std errors, p = 0.008) compared to congruent trials (Fig. 3-b). In contrast, in the passive task, we found a marginal main effect of *AOI* (χ^2^(3) = 7.0, p = 0.06), no main effect of *congruence* (χ^2^(1) = 0.47, p = 0.49), and no significant interaction between *congruence* and *AOI* (χ^2^(3) = 0.76, p = 0.87). In short, congruence seemed to affect pupil size but only during the emotion task (see SI for a complementary GLMM analysis using individual audio and video conditions).

We then investigated the temporal dynamics of the main effect of congruence in the emotion task. To do so, we analysed the effect of *congruence* on pupil dilation over time for each *AOI*. Specifically, we computed the median *pupil size* in 4-time intervals [(0, 2), (2, 4), (4, 6), (6, 8)] for each participant, each *congruence* condition (congruent vs incongruent), each *AOI*, and using only the data from the emotion task (Fig. 3-c). Then, we performed a GLMM analysis using *time*, *congruence* and *AOI* as predictors and participant-id as random factor. We found a main effect of *congruence* (χ^2^(1) = 20.4, p = 5.9e^−6^), a main effect of *time* (χ^2^(3) = 48.3, p = 1.8e^−10^) and no main effect of *AOI* (χ^2^(3) = 0.01, p = 0.99). However, we found no interaction between *time* × *congruence* (χ^2^(3) = 4.2, p = 0.23)*, time* × *AOI* (χ^2^(9) = 3.3, p = 0.95) or *AOI* × *congruence* (χ^2^(3) = 3.2, p = 0.36). These analyses suggest that the effect of congruence on pupil dilation is not localised in a specific temporal cluster—because *congruence* and *time* do not interact—but rather that there is a constant effect of congruence on pupil dilation across the trials in the emotion task.

Finally, because in our stimuli the audiovisual information mismatch is only present within the mouth area (the only area where our algorithmic transformation manipulates facial pixels), we investigated how the first fixation within the mouth—and other AOIs—influenced pupil dilation. To do this, for each AOI and for each trial, we discarded all the data before participants fixate for the first time that specific AOI. Using this reduced data, we performed a GLMM analysis testing for main effects of *congruence* after fixating each AOI (Fig. 3-d). The analysis revealed an interesting pattern of results. We found no significant main effect of congruence on *pupil size* after fixating for the first time within the eyes (χ^2^(1) = 1.7, p = 0.19), the rest of the face (χ^2^(1) = 1.4, p = 0.23) or the background (χ^2^(1) = 1.5, p = 0.21) areas. However, we found a significant main effect of *congruence* after participants fixated for the first time within the mouth area (χ^2^(1) = 6.6, p = 0.01). Specifically, incongruent trials triggered 0.06 ± 0.02 (std errors, p = 0.01) of pupil dilation compared to congruent trials. These results suggest that, in our data, the temporal precursor to the effect of *congruence* on *pupil size* is the first fixation within the mouth area.

## Discussion

Both visual and auditory features play a crucial role in emotional speech perception^4–9^. Although the neural mechanisms underlying the audiovisual integration of emotional signals have been thoroughly studied^1,10–14^, it remains unclear the physiological effects—such as the pupillary reactions—triggered by these mechanisms. That was the aim of the current study, to investigate if audiovisual emotional integration processes can be indexed by pupil dilation. Specifically, we studied how gaze patterns and pupil dilation reacted to emotional information mismatch in audiovisual speech. To do this, we created matching and mismatching audiovisual speech stimuli with digital signal processing algorithms able to manipulate the smiles seen in a person’s face and heard in their voice^31^. We then measured pupil dilation and gaze reactions while participants perceived the algorithmically manipulated stimuli in two tasks, a passive task (free viewing paradigm) and an emotional task (where participants’ attentional resources were directed to the emotional cues in the stimuli).

We found that pupil dilation can index emotional information mismatch in audiovisual speech. Previous findings had reported that incongruent features can trigger larger pupil dilation than their congruent counterparts, either when seeing a word and hearing a lexically incongruent sound^20^, or when observing emotionally incongruent scenarios (e.g., smiling while doing a negative action^21^). Similarly, pupillary reactions have been observed in reaction to arousing pictures ^25,27^ and vocalisations^29^. Here we show, for the first time, that pupil dilation can also index emotional information mismatch during the perception of audiovisual emotional speech. The fact that such pupillary reactions are observed in the literature for such a wide variety of tasks and stimuli suggests that these reactions are modality general and linked to the general processing of information mismatch.

One key novelty of the present work was to use real videos of people talking, rather than static pictures and co-occurring vocalisations^6,9,11,43,44^. This allowed us to investigate the dynamics of physiological reactions. Specifically, we investigated which gaze behaviors triggered pupillary reactions during emotional speech perception. In our data, the temporal precursor to pupil dilation seemed to be the first fixation inside the mouth area—exactly where our algorithmic model locally created the emotional incongruence in the stimuli. This suggests that the manipulated features in the stimuli, i.e., auditory and visual smiles, were processed jointly when actively extracting emotional information, and that information within foveal vision is dynamically integrated with the auditory signals over time during the perception of multimodal stimuli.

Our data also revealed a difference between passive and emotional tasks. Indeed, in our experiment, congruence only affected pupil size during the emotional task—when participants were extracting emotional information from the stimuli—but not during the passive task. One possibility is that, during the emotion task, participants’ attention was more importantly directed towards emotional features in the stimuli, which facilitated the perception of emotionally mismatching information. This interpretation reinforces previous findings that suggest that top-down emotion related attentional processes modulate pupillary reactions^29,39^, and also suggest that previous work on physiological reactions to emotional vocalisations may be influenced by the use of explicit emotional task sets^29,30,45^ as well as by the associated cognitive demands^35^. In this line, one possible interpretation of our result could be that, when participants are asked to extract emotional information from stimuli, information mismatch triggers higher levels of cognitive load because emotional information is ambiguous. This would subsequently trigger pupil dilation through the Locus Coeruleus-Norepinephrine system and, e.g., its associated activation of the ventral frontoparietal attention network^24,35^.

In any case, the fact that congruence only affected pupil dilation during the emotional task informs us on the causality of events triggering pupillary reactions. Indeed, the fact that focusing attentional resources on emotional features is enough to turn pupillary reactions on/off suggests that consciously recruited attentional mechanisms recruited for emotional processing can be the driving factor of pupillary reactions. Moreover, the fact that previous literature has reported that pupillary reactions to mismatching information can also be present in passive tasks^20,21^ suggests that, in some contexts, mismatching information is so salient that it has the potential to automatically hijack participants’ attentional resources later triggering pupillary responses. This opens the door to the investigation of the parameters that modulate pupillary responses during the perception of information mismatch (e.g., perceptual saliency, emotional context, cognitive load).

On another line, we also found a small effect indicating that participants’ gaze patterns changed subtly depending on information mismatch. Specifically, during the emotion task, participants’ gaze seemed to be less centred in the eyes during incongruent trials compared to congruent. However, because post-hoc tests did not hold after Bonferroni corrections for multiple comparisons, we do not interpret these effects in depth. In any case, while in our data these effects were small, they are consistent with recent literature studying gaze patterns during the processing of audiovisual information mismatch, which report similar gaze redirection to the eyes during congruent audiovisual trials^46^, as well as a spontaneous gaze redirection to congruent audiovisual information^3,43^. This study complements such findings with the use of dynamic audiovisual stimuli—rather than static face pictures.

This study has some limitations. First, in this experiment, the emotional task did not rely on any explicit behavioral response. Thus, although pupillary reactions were in line with predicted responses, which suggests that at least some part of the brain detected the incongruence in the stimuli, there is no way to know whether participants consciously perceived the stimuli as congruent/incongruent. Although we have shown in previous studies that both audio and visual manipulations are correctly recognized, and that the emotional information in visual and auditory channels interact during perception^31^, here we do not have that specific information on a trial by trial basis. This limitation was a trade-off to control for cognitive artefacts during explicit tasks, such as rating scales, that may ambiguate and trigger physiological responses on their own right by increasing motor demands^35^. Here, we preferred to have two strictly identical tasks in terms of motor responses, to control for this alternative—an experimental design choice which has, to our knowledge, not been thoroughly explored in previous literature.

Second, in the present experiment, task order was not randomized between participants. It may be that some of the effects we observe are influenced by task presentation order. We did this to not attract participants’ attention to emotional cues during the passive task, as this may bias gaze patterns with over-exploration of emotional features. However, this non-randomisation may influence our results. On the one hand, it may be that the non-randomisation explains the main effect of task on pupil dilation observed in Fig. 3-a. Indeed, pupil size may be higher at the beginning of the experiment because of the novelty of the experiment and the stimuli. On the other hand, the non-randomization may also explain the significant interaction observed between *task* and *AOI* for the gaze measures (Fig. 2-a). Indeed, gaze patterns may be influenced by task not only because of the underlying cognitive processes needed to decode emotional information, but also because of the novelty of the stimuli in the first part of the experiment. For these reasons, we do not interpret these results further. However, even if such familiarisation effects influenced gaze patterns and pupil reactions, they do not negate the fact that we observed an effect of congruence on pupil dilation in the emotion task. Rather, such limitations raise interesting questions about the specific contextual factors influencing pupil dilation during the processing of audiovisual emotional information mismatch. In the future, it would be interesting to randomize task-order, or to add an explicit emotional rating scale, to see e.g., whether physiological reactions increase with a motor response associated to emotional judgements, as has been done in previous pupillometry studies^29,30^.

Finally, some of the audio manipulations we used may not sound perfectly natural to external listeners. While we have validated the emotional valence of these manipulations, the artefacts in some stimuli may trigger different reactions in participants, such as attention shifts or discomfort. However, keep in mind that the stimuli in both congruent and incongruent conditions are *exactly* the same—the only thing that changes between conditions is the coupling between audio and video recordings. This implies that artefacts are constant between congruent and incongruent conditions. In other words, while there may be artefacts present in the stimuli, it is not clear why exactly the same artefacts would trigger pupil dilation in one case and not in another one. To control for such artefacts, and probably increase effect size, future experiments may want to use stronger emotional manipulations, which may include manipulations of e.g. vocal intonation^47,48^ in addition to auditory smiles.

In any case, we hope that the present study and methodologies will serve as a foundation to study the dynamic integration of audiovisual emotional speech and the associated physiological mechanisms. Furthermore, we hope the present experimental paradigm and results will be used to study disorders known for their impaired emotion processing, such as e.g., autism spectrum disorder^49^. This possibility seems of interest because the Locus Coeruleus-Norepinephrine system, and its associated activation of the ventral frontoparietal attention network, is proposed as an underlying mechanism of atypical attentional function in individuals with autism spectrum disorder^24^.

## Supplementary Information

Supplementary Information 1.

Supplementary Information 2.

## Data Availability

All data and resources including analysis scripts and stimuli are available on request. Please contact P.A.S. to get access to the resources.

## References

[1] Kreifelts B, Ethofer T, Grodd W, Erb M, Wildgruber D (2007). Audiovisual integration of emotional signals in voice and face: An event-related fMRI study. Neuroimage.

[2] Collignon O (2008). Audio-visual integration of emotion expression. Brain Res..

[3] Paulmann S, Pell MD (2011). Is there an advantage for recognizing multi-modal emotional stimuli?. Motiv. Emot..

[4] Baart M, Vroomen J (2018). Recalibration of vocal affect by a dynamic face. Exp. Brain Res..

[5] Föcker J, Gondan M, Röder B (2011). Preattentive processing of audio-visual emotional signals. Acta Psychol. (Amst).

[6] De Gelder B, Vroomen J (2000). The perception of emotions by ear and by eye. Cogn. Emot..

[7] Pourtois G, De Gelder B, Vroomen J, Rossion B, Crommelinck M (2000). The time-course of intermodal binding between seeing and hearing affective information. NeuroReport.

[8] De Gelder B, Böcker KBE, Tuomainen J, Hensen M, Vroomen J (1999). The combined perception of emotion from voice and face: Early interaction revealed by human electric brain responses. Neurosci. Lett..

[9] Vroomen J, Driver J, De Gelder B (2001). Is cross-modal integration of emotional expressions independent of attentional resources?. Cogn. Affect. Behav. Neurosci..

[10] Gao C, Weber CE, Shinkareva SV (2019). The brain basis of audiovisual affective processing: Evidence from a coordinate-based activation likelihood estimation meta-analysis. Cortex.

[11] Dolan RJ, Morris JS, De Gelder B (2001). Crossmodal binding of fear in voice and face. Proc. Natl. Acad. Sci. USA..

[12] Brunet-Gouet E, Decety J (2006). Social brain dysfunctions in schizophrenia: A review of neuroimaging studies. Psychiatry Res. Neuroimaging.

[13] de Jong JJ, Hodiamont PPG, Van den Stock J, de Gelder B (2009). Audiovisual emotion recognition in schizophrenia: Reduced integration of facial and vocal affect. Schizophr. Res..

[14] Lin Y, Ding H, Zhang Y (2020). Multisensory integration of emotion in schizophrenic patients. Multisens. Res..

[15] McGurk H, MacDonald J (1976). Hearing lips and seeing voices. Nature.

[16] Rosenblum LD, Schmuckler MA, Johnson JA (1997). The McGurk effect in infants. Percept. Psychophys..

[17] Colin C (2002). Mismatch negativity evoked by the McGurk-MacDonald effect: A phonetic representation within short-term memory. Clin. Neurophysiol..

[18] MorisFernandez L, Macaluso E, Soto-Faraco S (2017). Audiovisual integration as conflict resolution: The conflict of the McGurk illusion. Hum. Brain Mapp..

[19] MorisFernandez L, Torralba M, Soto-Faraco S (2018). Theta oscillations reflect conflict processing in the perception of the McGurk illusion. Eur. J. Neurosci..

[20] Renner LF, Włodarczak M (2017). When a dog is a cat and how it changes your pupil size: Pupil dilation in response to information mismatch. Proc. Annu. Conf. Int. Speech Commun. Assoc. Interspeech.

[21] Hepach R, Westermann G (2013). Infants’ sensitivity to the congruence of others’ emotions and actions. J. Exp. Child Psychol..

[22] Sabatinelli D (2014). The timing and directional connectivity of human frontoparietal and ventral visual attention networks in emotional scene perception. Neuroscience.

[23] Liddell BJ (2005). A direct brainstem-amygdala-cortical ‘alarm’ system for subliminal signals of fear. Neuroimage.

[24] Bast N, Poustka L, Freitag CM (2018). The locus coeruleus–norepinephrine system as pacemaker of attention—A developmental mechanism of derailed attentional function in autism spectrum disorder. Eur. J. Neurosci..

[25] Bradley MM, Miccoli L, Escrig MA, Lang PJ (2008). The pupil as a measure of emotional arousal and autonomic activation. Psychophysiology.

[26] Jessen S, Altvater-Mackensen N, Grossmann T (2016). Pupillary responses reveal infants’ discrimination of facial emotions independent of conscious perception. Cognition.

[27] Tamietto M (2009). Unseen facial and bodily expressions trigger fast emotional reactions. Proc. Natl. Acad. Sci. USA..

[28] Partala T, Surakka V (2003). Pupil size variation as an indication of affective processing. Int. J. Hum. Comput. Stud..

[29] Oliva M, Anikin A (2018). Pupil dilation reflects the time course of emotion recognition in human vocalizations. Sci. Rep..

[30] Cosme, G. *et al.* Pupil dilation reflects the authenticity of received nonverbal vocalizations. *Sci. Rep.* **11**, 1–14 (2021). 10.1038/s41598-021-83070-x PMC7880996 33580104

[31] Arias P (2018). Realistic transformation of facial and vocal smiles in real-time audiovisual streams. IEEE Trans. Affect. Comput..

[32] Ponsot E, Arias P, Aucouturier J-J (2018). Uncovering mental representations of smiled speech using reverse correlation. J. Acoust. Soc. Am..

[33] Barthel, H. & Quené, H. Acoustic-phonetic properties of smiling revised—Measurements on a natural video corpus. in *ICPhS* (2015).

[34] El Haddad, K. *et al.* Introducing AmuS: The amused speech database. in *Lecture Notes in Computer Science (including subseries Lecture Notes in Artificial Intelligence and Lecture Notes in Bioinformatics)* **10583 LNAI**, 229–240 (2017).

[35] van der Wel P, van Steenbergen H (2018). Pupil dilation as an index of effort in cognitive control tasks: A review. Psychon. Bull. Rev..

[36] Tamietto M, De Gelder B (2010). Neural bases of the non-conscious perception of emotional signals. Nat. Rev. Neurosci..

[37] Arias P, Belin P, Aucouturier JJ (2018). Auditory smiles trigger unconscious facial imitation. Curr. Biol..

[38] Arias, P., Bellmann, C. & Aucouturier, J. J. Facial mimicry in the congenitally blind. *Curr. Biol.* **31**, R1112–R1114 (2021). 10.1016/j.cub.2021.08.059 34637708

[39] De Gee JW, Knapen T, Donner TH (2014). Decision-related pupil dilation reflects upcoming choice and individual bias. Proc. Natl. Acad. Sci. USA..

[40] Olsen, A. The Tobii I-VT fixation filter. *Tobii Technology* **21**, 4–19 (2012).

[41] Gelman, A. & Hill, J. *Data analysis using regression and multilevel/hierarchical models*. (Cambridge university press, 2006).

[42] Zénon A (2017). Time-domain analysis for extracting fast-paced pupil responses. Sci. Rep..

[43] Rigoulot S, Pell MD (2012). Seeing emotion with your ears: Emotional prosody implicitly guides visual attention to faces. PLoS ONE.

[44] Paulmann S, Titone D, Pell MD (2012). How emotional prosody guides your way: Evidence from eye movements. Speech Commun..

[45] Partala T, Surakka V (2004). The effects of affective interventions in human-computer interaction. Interact. Comput..

[46] Rigoulot S, Pell MD (2014). Emotion in the voice influences the way we scan emotional faces. Speech Commun..

[47] Arias P, Rachman L, Liuni M, Aucouturier JJ (2021). Beyond correlation: Acoustic transformation methods for the experimental study of emotional voice and speech. Emot. Rev..

[48] Rachman L (2018). DAVID: An open-source platform for real-time transformation of infra-segmental emotional cues in running speech. Behav. Res. Methods.

[49] Saitovitch A (2013). Studying gaze abnormalities in autism: Which type of stimulus to use?. Open J. Psychiatry.

