# Author Correction: Pupil dilation reflects the dynamic integration of audiovisual emotional speech

**DOI:** 10.1038/s41598-023-33845-1

**Published:** 2023-04-25

**Authors:** Pablo Arias Sarah, Lars Hall, Ana Saitovitch, Jean-Julien Aucouturier, Monica Zilbovicius, Petter Johansson

**Affiliations:** 1 Lund University Cognitive Science, Lund University, Lund, Sweden; 2 STMS Lab, UMR 9912 (IRCAM/CNRS/SU), Paris, France; 3 School of Neuroscience and Psychology, Glasgow University, Glasgow, UK; 4 U1000 Brain Imaging in Psychiatry, INSERM-CEA, Pediatric Radiology Service, Necker Enfants Malades Hospital, Paris V René Descartes University, Paris, France; 5 Department of Robotics and Automation FEMTO-ST Institute (CNRS/Université de Bourgogne Franche Comté), Besançon, France

Correction to: *Scientific Reports* 10.1038/s41598-023-32133-2, published online 04 April 2023

In the original version of this Article Lars Hall and Petter Johansson were incorrectly affiliated with ‘Lab, UMR 9912 (IRCAM/CNRS/SU), Paris, France’. The correct affiliation is listed below.

Lund University Cognitive Science, Lund University, Lund, Sweden.

The original Article and accompanying Supplementary Information file have been corrected.

