# Supplementary material for: Pupil dilation reflects the dynamic integration of audiovisual emotional speech
Source: Sci Rep. 2023 Apr 4;13:5507. doi: 10.1038/s41598-023-32133-2 (PMC10073148; doi:10.1038/s41598-023-32133-2)
Supplement: Supplementary file 1 — Supplementary Information 1. [file 41598_2023_32133_MOESM1_ESM.docx]

Supplemental Information: Pupil dilation reflects the dynamic integration of audiovisual emotional speech

*Pablo Arias Sarah^1, 2,3,*^, Lars Hall^1^, Ana Saitovitch^4^, Jean-Julien Aucouturier^5^, Monica Zilbovicius^4^, Petter Johansson^1^*

*^1- Lund University Cognitive Science, Lund University, Lund, Sweden.^*

*^2- STMS Lab, UMR 9912 (IRCAM/CNRS/SU), Paris, France.^*

*^3- School of Neuroscience and Psychology, Glasgow University, Glasgow, UK.^*

*^4- U1000 Brain Imaging in Psychiatry, INSERM-CEA, Pediatric Radiology Service, Necker Enfants Malades Hospital, Paris V René Descartes University, Paris, France.^*

*^5- Department of Robotics and Automation FEMTO-ST Institute (CNRS/Université de Bourgogne Franche Comté), Besançon, France.^*

# Supplemental Figures

*Figure SI-1: Gaze Results. (a) Mean fixation duration for each AOI, and for each task. (b) Mean number of fixations for each task, for each AOI and for both congruent and incongruent conditions; error bars are 95% Confidence Intervals on the mean; Asterisks indicate statistically significant differences (p<Bonferroni*-α=*0.0125), “.”: indicate marginally significant differences (p<0.05).*

*Figure SI-2: Pupil size as a function of both audio condition and video condition (without grouping by congruence) for both the emotion (left) and passive (right) task..*

# Supplemental Analysis

## Gaze patterns

As a complementary analysis to the *number of fixation* analysis presented in the main text, we used *fixation duration* as an outcome in the GLMM models.

First, to study if gaze patterns were different across tasks, we performed a hierarchical GLMM analysis with *fixation duration* as an outcome, *task* (2 levels: passive, emotional) and *AOI* (4 levels : eyes, mouth, rest of face and background) as predictors, and *participant-id* as random factor with random slopes for task and *AOI* (Figure SI-1-a). We found a main effect of AOI (χ2(3)=118, p=2.2e^-16^), no main effect of task (χ2(1)=0, p=1) and a significant interaction between *AOI* and *task* (χ2(3)=44, p=1.2e^-9^ , figure SI-1-a). However, post-hoc t-tests comparing passive and emotion tasks for each AOI didn’t hold Bonferroni corrections for multiple comparisons (Bonferroni-α = 0.0125, eyes : t(29)=0.9, p=0.35; mouth : t(29)=2.3, p=0.02; rest of face : t(29)=2.0, p=0.05; background : t(29)=0.7, p=0.48). Second, we investigated if *congruence* affected *fixation duration*. To do so, we performed a GLMM analysis to test for effects of *AOI* (4 levels: eyes, mouth, rest of face, background) and *congruence* (2 levels: congruent, incongruent) using *fixation duration* as an outcome (figure SI-2-b). We used participant number as a random factor with a random slope for *AOI*. In line with the main analyses, we found a main effect of *AOI* both in the emotion (χ2(3)=111, p=2.2e-16) and in the passive task (χ2(3)=120, p=2.2e-16), no main effect of *congruence* in any task (χ2(3)<1, p>0.9), and a significant interaction between *congruence* and *AOI* in the emotion task (*congruence* x *AOI*: χ2(3) = 9.7, p=0.02), but not in the passive task (χ2(3) = 1.2, p= 0.73). In short, we observed very similar results as the ones observed for *fixation duration* (Main figure 2) when using *fixation duration* as an outcome*.*

## Pupil data

As a complementary analysis to the main GLMM analysis of pupil size in the main manuscript (main figure 3), we analysed pupil data using GLMMs but using both *video condition* and *audio condition* as predictors, and *participant_id* as random factor (Figure SI-2). In line with main results, in the emotion task we found no main effect of *audio condition* (χ2(1)=0.5, p=0.45), no main effect of *video condition* (χ2(1)=0.49, p=0.48) and a significant interaction between audio and video effects (χ2(1)=6.0, p=0.01). Conversely, in the passive task, we found no main effect of either *audio condition* (χ2(1)=1.0, p=0.30) or *video condition* (χ2(1)=1.9, p=0.16), and no significant interaction between *audio* and *video condition* (χ2(1)=0.72, p=0.39)

# Supplemental Methods

## Supplemental stimuli information

We used the following sentences to create the stimuli for the main experiment

| Original sentence | English Translation |
| --- | --- |
| On m’a dit que les montagnes sont belles à cette période de l’année, j’espère pouvoir les voir. | I was told that the mountains are beautiful at this time of the year, I hope I’ll be able to see them. |
| Je suis toujours en retard au travail le lundi matin, au même temps, c’est normal, j’ai trop de mal à me lever. | I’m always late for work on Monday morning, at the same time, it’s not surprising, I have a hard time waking up. |
| Je suis passioné de cinéma, de musique et de théâtre mais ce que je préfère c’est aller voir des concerts | I am passionate about cinema, music and theater, but what I prefer is to attend concerts. |
| J’aime beaucoup nos vacances de noël en famille, j’adore retrouver ma grande mère, mes oncles et mes cousins | I like ta lot Christmas holidays with my family, I love seeing again my grandmother, my uncle and my cousins. |
| Leurs amis organisent un soirée déguisé la semaine prochaine, je pense que je vais y aller en Spiderman | Their friends are organizing a costume party next week, I think I’m going to dress up as Spiderman. |
| Elle se promène dans la forêt tous les dimanches après-midi, quand elle rentre, elle fait la sieste | She often goes walking on Sunday afternoon, when she comes back, she takes a nap. |
| L’atmosphère là-bas est vraiment sympathique, j’aimerais pouvoir y rester toute la semaine. | The atmosphere there is really nice, I would love to be able to stay there the whole week. |
| Un nouveau restaurant japonais vient d’ouvrir de l’autre côté de la rue, c’est de la cuisiune fusion japonaise, ça a l’air très bon. | A new Japaneese restaurant just opened in the other side if the street, it’s Japanese fusion cuisine, it looks very good |
